# A potential histoplasmosis pathogenesis mechanism mediated by a lectin of *Histoplasma capsulatum* with affinity to β-galactose, favoring intravascular hemagglutination

**DOI:** 10.1371/journal.pntd.0014105

**Published:** 2026-03-10

**Authors:** Ivan Ramos-Martínez, Eugenia Flores-Robles, Gabriela Rodríguez-Arellanes, Edgar Zenteno, Armando Pérez-Torres, Maria Lucia Taylor

**Affiliations:** 1 Unidad de Micología, Departamento de Microbiología-Parasitología, Facultad de Medicina, Universidad Nacional Autónoma de México (UNAM), Mexico City, Mexico; 2 Departamento de Bioquímica, Facultad de Medicina, UNAM, Mexico City, Mexico; 3 Departamento de Biología Celular y Tisular, Facultad de Medicina, UNAM, Mexico City, Mexico; FIOCRUZ: Fundacao Oswaldo Cruz, BRAZIL

## Abstract

Ascomycetes belonging to the *Histoplasma capsulatum* species complex can cause severe manifestations in patients with disseminated histoplasmosis. Based on data from hemagglutination assays and histopathological findings related to *H. capsulatum*, we investigated their potential role in the disseminated intravascular hemagglutination events. Human erythrocyte hemagglutination was produced with *H. capsulatum* yeasts of 10 isolates/strains, belonging to different phylogenetic species and lineages. Hemagglutination assays were inhibited by pre-incubating their yeasts with β-galactose, ratifying previously published data for a unique *H. capsulatum* clinical strain. This lectin was partially purified through affinity chromatography using erythrocyte stroma, and its hemagglutinating activity was confirmed. Results underlined the consistent involvement of a β-galactose-binding lectin of *H. capsulatum* yeasts in hemagglutination assays by using genetically distinct *H. capsulatum* species, which support its conserved origin. Serendipitous findings in histopathological sections of human and bat tissues supported that *H. capsulatum* yeasts adhered to erythrocytes, consistent with intravascular hemagglutination. Hence, in hosts with disseminated histoplasmosis and sepsis, we hypothesized that this *H. capsulatum* lectin may favor an intravascular hemagglutination, which could occur concomitantly with a procoagulant state, aggravating hypoxia and leading to disseminated intravascular coagulation, a lethal complication.

## Author summary

Histoplasmosis, caused by *Histoplasma capsulatum* complex, is a respiratory-systemic mycosis prevalent in the Americas. This disease can display life-threatening acute pulmonary and disseminated clinical forms. Here, we hypothesized that the hemagglutinating activity mediated by the β-galactose-binding lectin present on yeasts of genetically distinct *H. capsulatum* isolates/strains could be involved in the fatal complications of septic patients, such as disseminated intravascular coagulation.

## Introduction

Histoplasmosis is the most important systemic mycosis caused by dimorphic fungi. It is produced by the *Histoplasma capsulatum* species complex. The systematics of this fungal complex is being rearranged. Currently, 14 phylogenetic species have been described across five continents based on compiled data regarding *H. capsulatum* classifications [1]. Histoplasmosis can affect both immunocompetent and immunocompromised individuals. Among histoplasmosis clinical manifestations, the disseminated intravascular coagulation (DIC) with associated complications stands out for its severity [2,3]. However, the pathophysiological mechanisms that trigger DIC in this context are poorly understood.

Previous publications using a particular *H. capsulatum* clinical isolate, now classified as LAm A2 phylogenetic species, have reported yeasts binding to human erythrocytes, contributing to hemagglutination, which was competitively inhibited by adding β-galactose [4,5]. *Histoplasma capsulatum* was described as capable of binding to a glycosphingolipid, lactosylceramide (Galβ1–4Glcβ1-1Cer), which is present in the epithelial tissue of some mammalian hosts, including humans [6]. *Histoplasma capsulatum* yeasts were also found to adhere to murine laminin, a major glycoprotein of basal lamina, where adherence was mediated by a fungal 50-kDa protein [7]. This observation is consistent with the present report and supported by Jin et al. [8], revealing the fact that laminin contains high concentrations of galactosyl residues.

Few reports associated fungal lectins with the pathogenesis of respiratory mycoses. Therefore, it is interesting to note that a yeast phase-specific protein of *H. capsulatum*, known as Yps3p, has affinity for chitin, as reported by Bohse and Woods [9]. Furthermore, a review published by Pitangui et al. [10], highlights a lectin from *Paracoccidioides brasiliensis* that is involved in fungal virulence and host immunomodulatory activity.

Here, we aimed to determine the presence of a β-galactose-binding lectin on the *H. capsulatum* yeast cell-wall across different phylogenetic species and lineages, isolated from distinct host origins in the American continent. In addition, we propose the role of this lectin in histoplasmosis pathogenesis, based on circumstantial evidence from *Histoplasma*-infected hosts that suggests a close link between fungal yeast intravascular hemagglutination and DIC complications, in patients with disseminated histoplasmosis.

## Methods

### Ethics statement

*Postmortem* studies of the patient followed the Rokitansky autopsy protocol, as approved by the Ethics Committee of the “Manual Gea Gonzalez” Hospital, Ministry of Health, Mexico. The autopsy was registered with the code A-13–04. Hospital autopsy consent was based on the Mexican Official Guide (NOM 168-SSA1–1998) and the American College of Pathologists guideline. Regarding the ethics statement for bat capture, the mandatory requirements of the Animal Care and Use Committee of the Universidad Nacional Autónoma de México (UNAM) and the Mexican Official Guide (NOM 062-ZOO-1999) were followed.

Human (EH-46, EH-53) and bats (EH-315, EH-374, EH-378, EH-672H, EH-696P) *H. capsulatum* isolates/strains studied are deposited in the “Colección de Cepas de *Histoplasma capsulatum*” of the “Laboratorio de Inmunología de Hongos-Unidad de Micología, Departamento de Microbiología-Parasitología, Facultad de Medicina,” UNAM (http://www.facmed.unam.mx/histoplas-mex/colcepas.html). The collection is registered in the World Data Centre for Microorganisms (WDCM), number LIH-UNAM WDCM817 (http://www.wfcc.info/ccinfo/index.php/strain/display/817/fungi/). Strains G-186B, G-217B, and Downs from the American Type Culture Collection (ATCC) were used as references.

Fungal yeast phase was induced at 37°C in brain-heart infusion broth (Bioxón; Becton Dickinson, Mexico City, Mexico) supplemented with 0.1% L-cysteine and 1% glucose. The pellet containing viable *H. capsulatum* yeasts was obtained by centrifuging at 800 x *g* for 10 min.

For the hemagglutination assay, 1 x 10^6^ human O erythrocytes were incubated with 5 x 10^6^ yeasts in phosphate-buffered saline (PBS) at 37°C for 1 h. In all assays, PBS buffer without yeast was used as a negative control and a lectin of *Cherax quadricarinatus* (hemolymph) was used as a positive control.

The generated hemagglutinating units (HAU) were calculated as the highest serial dilution showing hemagglutination. The inhibition of hemagglutination was achieved by pre-incubating *H. capsulatum* yeasts with different concentrations of β-galactose (Sigma Chemical Co., St. Louis, MO, USA), as described by Taylor et al. [4].

Affinity chromatography was used to purify the β-galactose-binding lectin from two representative *H. capsulatum* isolates, one from a human clinical case (EH-53) and the other from a naturally infected bat (EH-374). Fungal yeasts were lysed using a sonicate apparatus (Soniprep 150 Henserson Biomedical, UK), using 10 µm amplitude for 2 min with 20 s intervals at room temperature, and a frequency of 23 KHz, in the presence of PBS supplemented with protease inhibitors. Lysed yeasts or yeast culture-supernatants were loaded on a chromatographic column with human O erythrocyte stroma-Sephadex G-25 (Pharmacia Chemicals, Upsala, Sweden) column. The non-retained fraction (NRF) and the retained fraction (RF) were eluted with PBS and 3% acetic acid, respectively. They were collected in tubes containing 1 ml each. All RFs collected were stopped when their optical density was no longer detectable and, after, these RFs were dialyzed in PBS. Absorbance at 280 nm was monitored in both fractions. Protein concentrations of both fractions were determined using the Lowry method [11], and subsequently, their hemagglutinating activity was tested.

Infected-host tissue samples, either from a human patient or a captured bat, were tested by nested-PCR reaction, using the amplification of the *Hcp10*0 gene, which is a specific molecular marker for diagnosing *H. capsulatum* [12]. Histological procedures were performed on paraffin sections (4 µm) using neutral buffered formalin-fixed tissues from the necropsy samples of the woman patient and from the liver of a randomly captured bat. Samples were stained with Grocott’s modification of Gomori’s Methenamine Silver histochemical method [13]. Histopathological preparations were analyzed using a BX50 Olympus microscope (Olympus Corporation of the Americas, Center Valley, PA, USA) equipped with a digital camera and Infinity Analyze software, v6.3.0.

## Results

This paper tested 10 *H. capsulatum* isolates/strains obtained from naturally infected mammalian hosts (humans and bats), listed in Table 1.

**Table 1:** Yeast hemagglutination assays with human erythrocytes.

Isolates/ Strains	Mammalian hosts	Phylogenetic species^1^	HAU^2^	Specific activity^4^	β-galactose inhibition (mM)
EH-46	Human	LAm A2	2	3.3	200
EH-53	Human	LAm A2	2	5.3	50
EH-315	Bat	NAm 3	8	4.5	1.5
EH-374	Bat	LAm A1	32	107.0	100
EH-378	Bat	LAm A2	4	2.9	1.5
EH-672H	Bat	Lineage	4	3.1	50
EH-696P	Bat	Lineage HI53	2	5.2	1.5
G-186B^3^	Human	Lineage H83	2	7.2	200
G-217B^3^	Human	NAm 2	8	6.1	50
Downs^3^	Human	NAm 1	4	14.92	1.5

^1^Reported in [14–16]; ^2^Hemagglutinating unit; ^3^Reference strains from the ATCC; ^4^Specific activity is defined through HAU divided by the protein concentration (mg/ml). Hemagglutination assays were set up in triplicate. For the assays, PBS buffer and *C. quadricarinatus* hemolymph were used as positive and negative controls, respectively.

The presence of the β-galactose-binding lectin on the yeast cell-wall of each cultured *H. capsulatum* studied was inferred by hemagglutination assays using human erythrocytes and expressed as HAU; also, hemagglutination was inhibited once *H. capsulatum* yeasts were incubated with β-galactose (Table 1).

**Fig 1:**
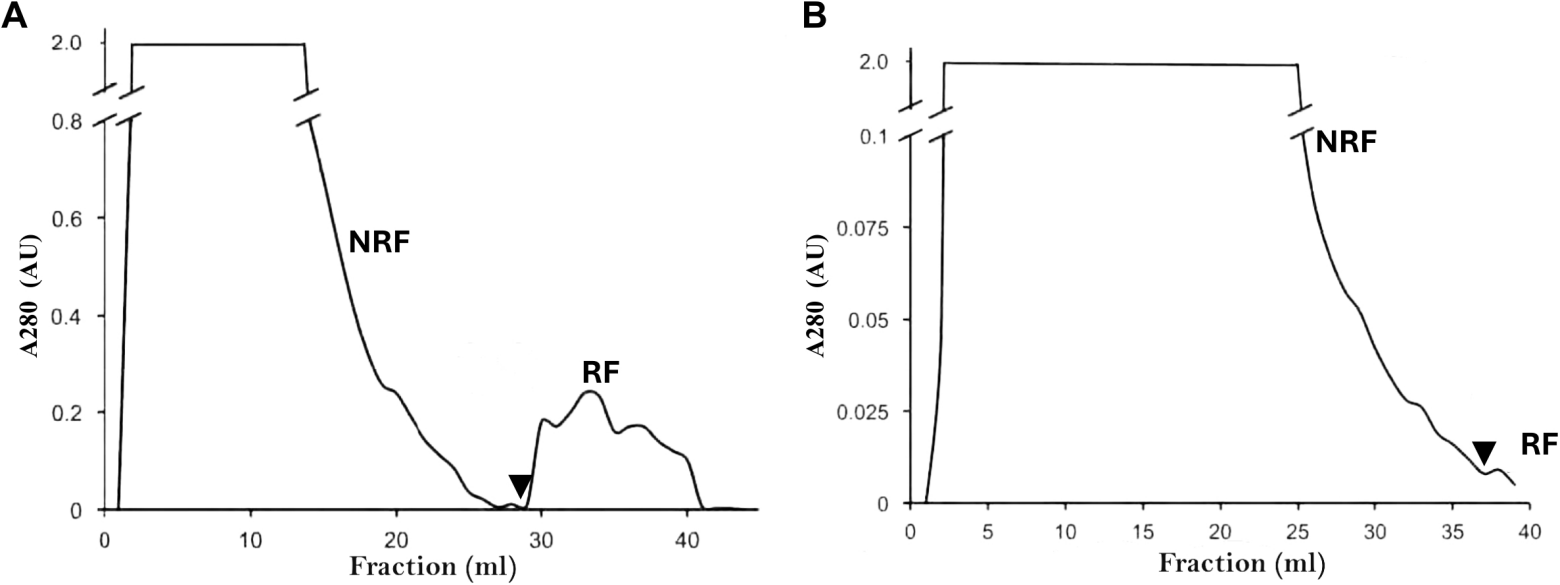
Affinity chromatography with human erythrocyte stroma. **(A)** Soluble components of the yeast-culture medium. **(B)** Yeast-homogenate from the *H. capsulatum* EH-53 isolate. AU = Adsorption Units at 280 nm. Arrowheads indicate the start of elution with 3% acetic acid.

*Histoplasma capsulatum* lectin was obtained from the yeast-culture medium (soluble fraction) and the yeast-homogenate of the EH-53 and the EH-374 *H. capsulatum* isolates, by using affinity chromatography with human erythrocyte stroma. Fig 1 shows a representative chromatogram of the EH-53 isolate, in which the retained fraction eluted off the chromatographic column was monitored at 280 nm and tested for hemagglutinating activity.

The RFs generated with soluble and homogenate fractions, using either the EH-53 or the EH-374 isolates, displayed a variable range of protein concentrations and produced hemagglutination. The RF of the yeast-soluble fraction from the EH-53 isolate showed a specific activity of 20 (8 HAU/0.04 mg/ml of protein/), while the RF corresponding to its yeast-homogenate showed a specific activity of 50 (4 HAU/0.08 mg/ml of protein). In addition, the RF of the yeast-soluble fraction from the EH-374 isolate showed a specific activity of 200 (4 HAU/0.02 mg/ml of protein), while the RF corresponding to its yeast-homogenate showed a specific activity of 66 (4 HAU/ 0.06 mg/ml of protein).

Results using the RFs collected from both *H. capsulatum* isolates (EH-53 and EH-374) developing hemagglutination, either with their soluble fractions of the yeast-culture medium or with their yeast-homogenate fractions, support the fact that the β-galactose-binding lectin is a fungal cell-wall protein and that it can be solubilized in the medium. In contrast, all NRFs did not develop hemagglutination in any assay.

**Fig 2:**
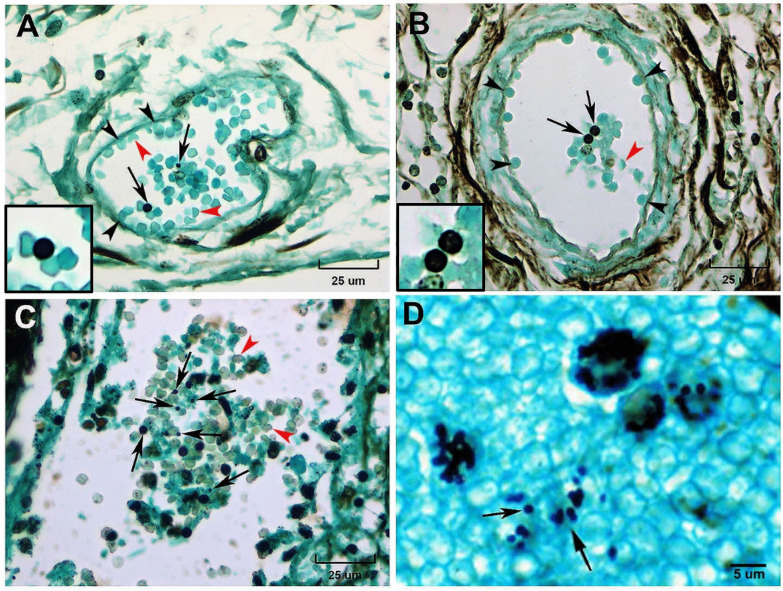
Intravascular hemagglutination mediated by *H. capsulatum* yeasts in a patient diagnosed *postmortem* with disseminated histoplasmosis and in a randomly captured infected bat. Histopathological findings in the cecum and ileum sections revealed hemagglutination formed by clusters of erythrocytes containing yeasts (**A-C**, black arrows). Abnormal adhesion of erythrocytes to the vascular endothelium was observed (**A** and **B**, black arrowheads). The presence of schistocytosis was observed, based on the altered erythrocyte morphology (**A-C**, red arrowheads). In a liver histological section of a randomly captured *H. capsulatum*-infected bat, fungal yeasts adhering to erythrocytes were also observed **(D**, black arrows**)**. Grocott-Gomori methenamine silver histochemistry. Bars: A-C = 25 µm; D = 5 µm.

The hemagglutinating ability of *H*. *capsulatum* yeasts was observed in the histopathological findings of necropsy samples of the woman patient who died with disseminated histoplasmosis, diagnosed *postmortem*. The previous patient´s clinical data referred to septic shock and DIC after abdominal pain syndrome, which evolved to an acute abdomen due to cecum perforation. The presence of extracellular *H. capsulatum* yeasts adhered to erythrocytes was found intravascularly in cecum and ileum samples of her necropsy material (Fig 2A-C). In the ileum and cecum, a single yeast attached to one or two erythrocytes was observed in 70% of random microscopic fields. The same hemagglutination event observed in the submucosa and serosa of the cecum and ileum was also observed in a distant organ of the patient, the vessel of the cerebellar granule cell layer (see S1 Fig). Unusual adhesion of erythrocytes was observed in the intestinal vascular endothelium (Fig 2A and B). Furthermore, erythrocyte fragmentation (schistocytosis) can be observed (see Fig 2A and B), highlighting that these erythrocyte alterations could be associated with inflammation, sepsis, hypoxia, and DIC. Interestingly, yeasts adhered to erythrocytes within a blood vessel were also identified in liver samples of a randomly captured *H. capsulatum*-infected bat (Fig 2D).

## Discussion

Hemagglutinating activity mediated by *H. capsulatum* yeasts has been reported previously by our research group [4,5], suggesting a potential fungal pathogenicity mechanism that may contribute to histoplasmosis pathogenesis. This mechanism remained unexplored for a long time; although it was recognized in a particular article published by Vargas et al. [17], which focused on histoplasmosis damage. This report presented histopathological findings in a disseminated histoplasmosis clinical case with DIC and septic shock, revealing the intravascular presence of extracellular *H. capsulatum* yeasts adhered to erythrocytes [17]. Given the importance of this issue, our group has incorporated novel data supporting the role of a β-galactose-binding lectin that may contribute to a procoagulant state and sepsis in some histoplasmosis-associated complications.

The presence of this lectin in several isolates/strains of infected humans and bats from North and Central America supports the fact that it is a conserved protein, suggesting that it may fulfill an essential biological function. It is worth noting that hemagglutination assays with *H. capsulatum* yeasts revealed different hemagglutinating units (see Table 1); however, these differences may be due to β-galactose concentrations and the affinity of the *H. capsulatum* lectin of each fungal isolate, suggesting minor changes in the lectin carbohydrate-binding domain.

It has been reported that *H. capsulatum* yeasts of the EH-53 isolate were able to hemagglutinate erythrocytes of the human ABO group, and no significant differences were detected in the respective hemagglutination results [4], even though the position of the β-galactose is variable among erythrocytes of the ABO group.

An interesting interaction between *H. capsulatum* and host components was previously studied by Jimenez-Lucho et al. [6], who found that lactosylceramide participates in a host inflammatory pathway by activating NADPH oxidase, leading to oxidative stress and neutrophil activation [18]. Possibly, either hemagglutination by *H. capsulatum* yeasts or lactosylceramide binding is mediated by the same molecule studied here. This could stimulate the exploration of a new mechanism of inflammation in disseminated histoplasmosis, with repercussions for host immunomodulation mediated by the β-galactose lectin of *H. capsulatum*. Likewise, this type of interaction should be tested for new therapeutic approaches.

Additionally, Taylor et al. [4] have suggested that *H. capsulatum* yeasts can bind to other extracellular matrix components, such as chondroitin-sulfate A and C, as well as heparan sulfate, showing the highest affinity for chondroitin-sulfate C due to the presence of N-acetyl-galactosamine. These findings support the binding of fungal yeasts to the extracellular matrix of subepithelial regions of the tracheal lamina propria and the adventitia, as experimentally demonstrated in bat and mouse models by Suárez-Álvarez et al. [19], which could lead to the dissemination of *H. capsulatum* to adjacent tissues.

In view that both *H. capsulatum* isolates (EH-53 and EH-374) developed hemagglutination activity either in the soluble fractions of the yeast-culture medium or in its yeast-homogenate fraction, it is possible to propose that the β-galactose-binding lectin is a fungal cell-wall protein and can be solubilized in the medium.

In rare cases of histoplasmosis, erythrocyte adhesion to the vascular endothelium has been observed, as shown in Fig 2A and B of the present paper. In histoplasmosis, mainly in immunocompromised and septic patients, hemagglutination-associated ischemia could favor the development of a procoagulant state. Overall, sepsis progresses to septic shock and disseminated coagulation, leading to multiple organ dysfunction and, finally, death. These threatening pathogenic manifestations have been reported in the histoplasmosis literature [3,4,20–24]. In theory, we considered that similar clinical manifestations could be correlated with different pathogenic events described in disseminated clinical cases. Here, we refer to intravascular hemagglutination caused by the *H. capsulatum* β-galactose-binding lectin that could overlap with other mechanisms, such as DIC, which has been observed in disseminated histoplasmosis, both in HIV [3,4,20,25] and non-HIV patients [17,18,26]. Hemagglutination mediated by the β-galactose lectin differs from the hemophagocytic syndrome [24–29], which has also been described in histoplasmosis. Hemophagocytic syndrome is characterized by an uncontrolled T cell response and subsequent macrophage activation, leading to the phagocytosis of hematopoietic cells and the production of excessive inﬂammatory cytokines [30]. Although the β-galactose lectin has been reported to bind to the murine macrophage membrane by their galactosylated surface molecules [31], this event is not related to the hemophagocytic syndrome, since no erythrocyte phagocytosis was observed.

The complete purification of the β-galactose-binding lectin of *H. capsulatum* is a crucial drive for designing experimental strategies to demonstrate that this lectin has a potential role as “an aggravating co-factor”. In disseminated histoplasmosis, it is probably necessary to form septic stages driven by systemic inflammation to promote hemagglutination mediated by the β-galactose lectin, which could aggravate hypoxia secondary to erythrocyte microcirculatory obstruction. This could favor endothelial dysfunction, which is observed in DIC. In fact, Fig 2A and B shows suggestive images of endothelial dysfunction mediated by erythrocytes’ adhesion to the intestinal vascular endothelium.

## Conclusion

This study highlights the importance of a β-galactose-binding lectin expressed on distinct phylogenetic species of *H. capsulatum*. The presence of this lectin on the yeast cell-wall supports its potential involvement in intravascular hemagglutination, which could occur together with DIC. The forthcoming definition of the physical-chemical characteristics of the β-galactose lectin and its subsequent purification will enable the use of robust tools to identify its genetic sequence and better characterize its role in histoplasmosis pathogenesis.

## Supporting information

S1 Fig Binding of *H. capsulatum* yeasts to erythrocytes in different histopathological sections of a patient with disseminated histoplasmosis. (TIF)
